# A handy balloon for total extraperitoneal repair of inguinal hernia

**DOI:** 10.4103/0972-9941.41952

**Published:** 2008

**Authors:** Vishwanath Golash

**Affiliations:** Department of Surgery, Sultan Qaboos Hospital, P.O. Box: 98, Salalah, 211, Sultanate of Oman

**Keywords:** Balloon, extraperitoneal space, inguinal hernia, laparoscopy

## Abstract

**Background:**

The creation of extraperitoneal space with the help of balloon is helpful in the learning curve of total extraperitoneal repair of inguinal hernia. But the cost of the balloon with other disposable items is one of the limiting factors in the popularity of this operation. By using a homemade balloon, nondispoasable ports and suturing the mesh we have been able to reduce the cost considerably. We would like to present our technique of making the balloon.

**Materials and Methods:**

Between September 2005 and November 2006 we operated 65 consecutive patients of inguinal hernia using the balloon made from the ′video camera drape′. The drape was cut and fixed on a 10 mm suction irrigation cannula. The suction irrigation cannula was used as an obturator as well to inflate the balloon in the extraperitoneal space.

**Results:**

We succeeded in creating the extraperitoneal space in all patients with the help of this balloon without any complications and conversion to open. There was no leak and bursting of the balloon.

**Conclusion:**

It is cheap, strong, sterile and readily available material for making the balloon for creating the extraperitoneal space in the laparoscopic repair of the inguinal hernia.

## INTRODUCTION

The total extraperitoneal approach is the method of choice in the laparoscopic repair of the inguinal hernia. It is technically a difficult procedure and creation of extra peritoneal space with the help of balloon is helpful in the learning curve. The cost of disposable items is other limiting factors in the popularity of this approach. There have been several innovations in technique and instruments to make it technically easier and to lower the cost of operation. By using a homemade balloon, nondispoasable ports and with our technique of fixing the mesh with suture we have made the technique cost-effective.[1]

## MATERIALS AND METHODS

Between September 2005 and November 2006, the laparoscopic repair of inguinal hernia was done on consecutive 65 patients using a homemade balloon for creating the extraperitoneal space. There were five recurrent hernias, 15 bilateral hernias and 45 unilateral inguinal hernias. All patients were male in age group of 18-85 years. The perforated end of the ′video camera drape′ with its attached sticky tape (Microtec Medical Inc, US Patent No. 5,061,246) was fixed on a 10 mm suction irrigation cannula (Aesculap GF 325 R 10mm, 400 mm length), 15 cm from its tip as shown [Figure 1A]. The other end of the video camera drape was doubly tied with 2-0 vicryl suture close to the tip of the cannula and excess length was cut off [Figure 1B]. This created a balloon on the shaft of the suction irrigation cannula (obturator), 15 cm long and 10 cm wide without inflation [Figure 1C, D]. The balloon was tested with air insufflations before introduction. We found the video camera drape a strong material and were able to hold 1000 cc air without bursting and leaking. After finger dissection it was easy to advance the balloon on an obturator in the extraperitoneal space until it touches the pubic bone. The 10 mm suction irrigation cannula has rounded smooth tip, which is atraumatic when introduced in the extraperitoneal space also does not punctures the material of video camera drape. It has a one-way trumpet valve at the other end and does not allow air to leak [Figure 2A, B].

**Figure 1A:**
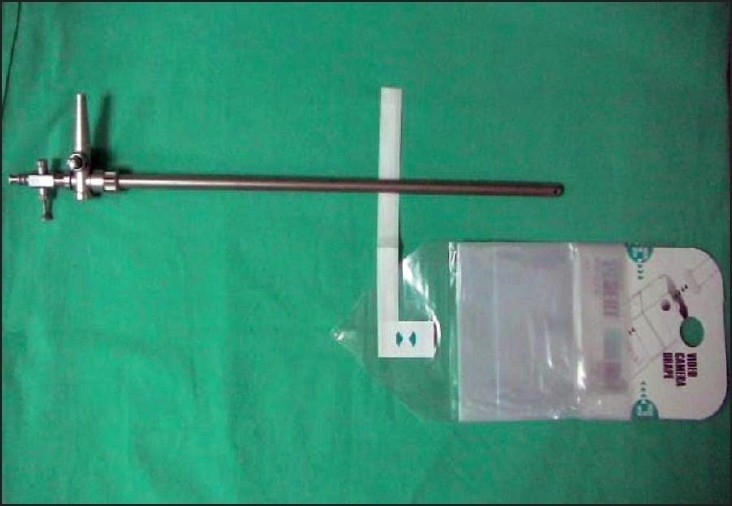
Perforated end of video camera drape to be fixed on suction irrigation cannula

**Figure 1B:**
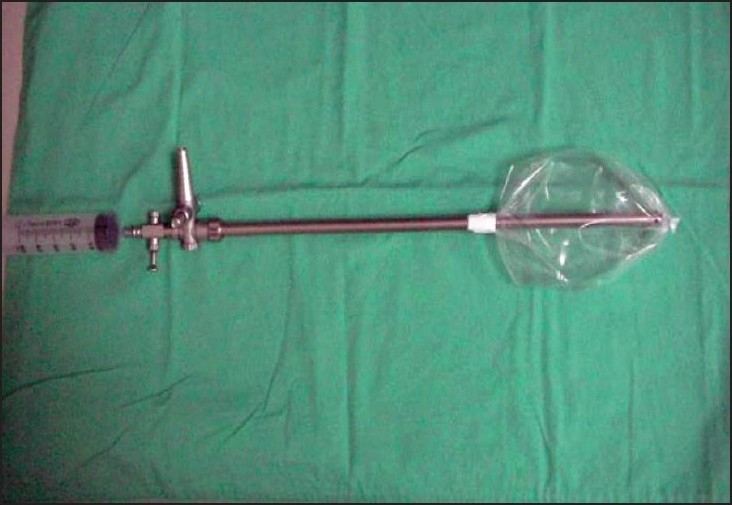
End of drape tied to suction cannulab

**Figure 1C:**
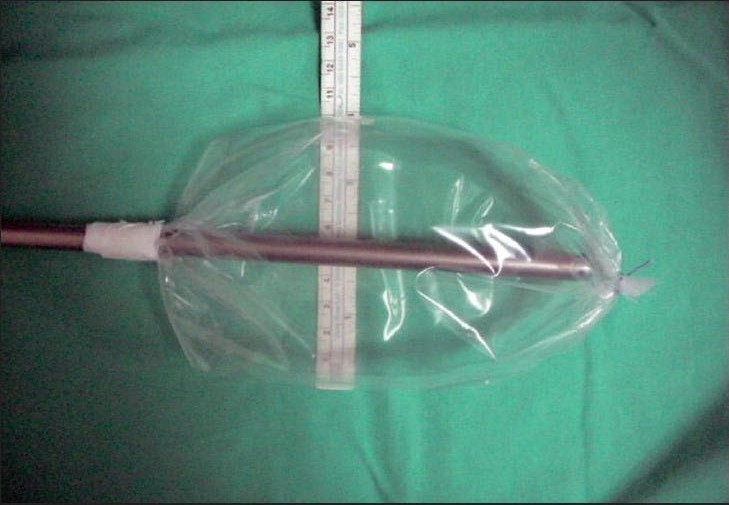
Ten cm wide balloon

**Figure 1D:**
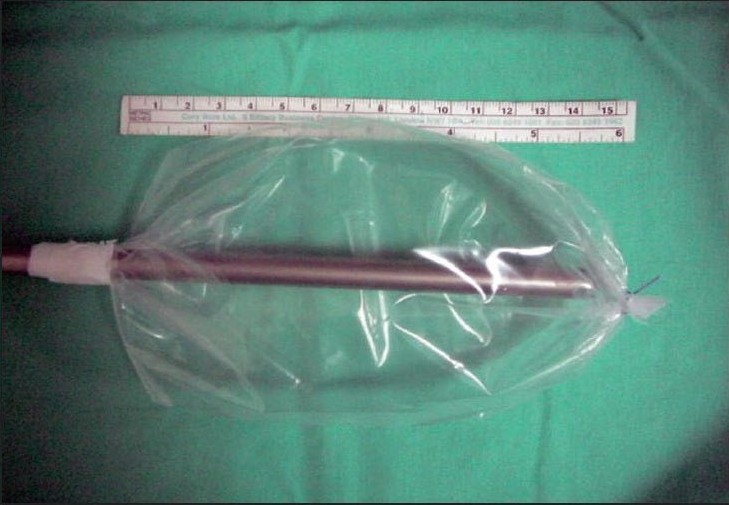
Fifteen cm long balloon

**Figure 2A:**
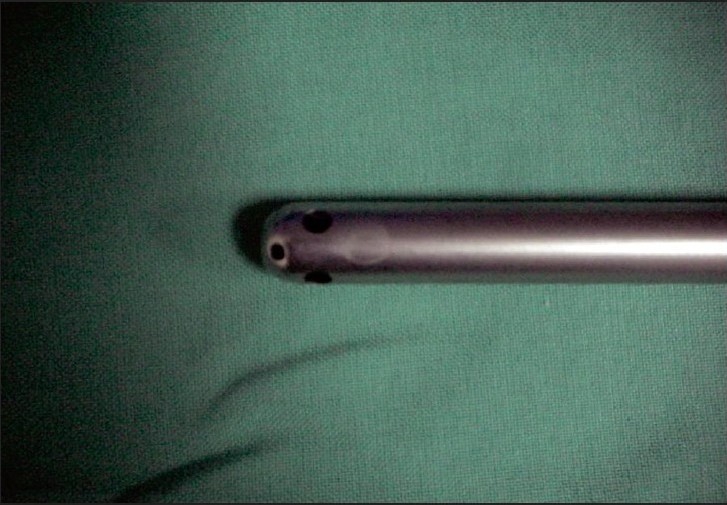
Smooth end of the suction irrigation cannula

**Figure 2B:**
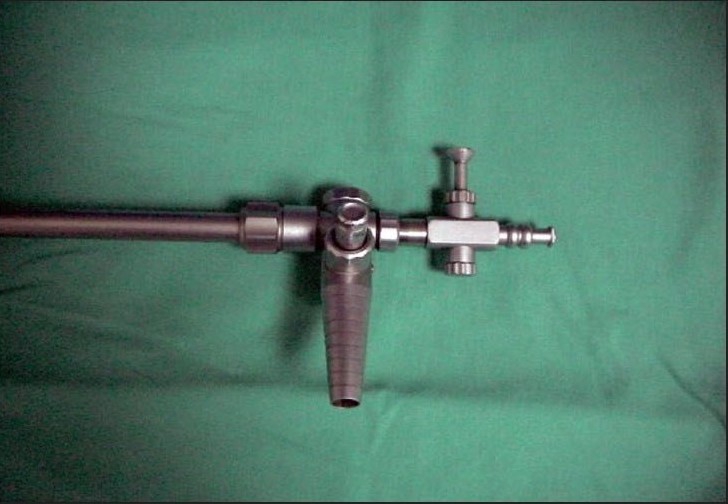
Trumpet end of the suction irrigation cannula

## RESULTS

We succeeded in creating the extraperitoneal space in all patients with the help of this balloon without any complications and conversion to open. There was no leak and bursting of the balloon.

## DISCUSSION

With increasing experience in the technique the extraperitoneal space can be created without the use of balloon but it is helpful to use balloon in the learning curve and has been shown to reduce the conversion rate.[2,3] To make it cost-effective several modifications of locally made balloons using surgical gloves etc. have been tried successfully.[4,5] In our balloon, the obturator (suction irrigation cannula) is helpful in introducing, positioning and inflating the balloon in the appropriate area. 

## CONCLUSIONS

It is a strong, cheap and readily available material to make a balloon for creating the extraperitoneal space.
